# The cutting-edge roles of lasers in endodontics: A bibliometric and scientometric analysis of the 100 most-cited articles

**DOI:** 10.1007/s10103-024-04163-3

**Published:** 2024-08-16

**Authors:** Sıla Nur Usta, Pablo Betancourt, Alper Ceylan, Cangül Keskin

**Affiliations:** 1 Department of Endodontics, Gulhane Faculty of Dentistry, University of Health Sciences, Ankara, Turkey; 2 Center for Research in Dental Sciences (CICO), Endodontic Laboratory, Faculty of Dentistry, Universidad de La Frontera, Temuco, 4780000 Chile; 3 Department of Integral Adultos, Faculty of Dentistry, Universidad de La Frontera, Temuco, 4780000 Chile; 4 Department of Endodontics, Faculty of Dentistry, University of Ondokuz Mayıs, Samsun, Turkey

**Keywords:** Bibliometrics, Diode laser, Endodontics, Laser, Photodynamic therapy

## Abstract

Purpose: This bibliometric and scientometric analysis aimed to delve into the forefront roles of lasers in endodontics from 1990 to 2024. Methods: A comprehensive electronic search was conducted using “Clarivate Analytics Web of Science, All Databases” to retrieve the most-cited articles pertaining to the topic. These articles were then ranked in descending order according to their citation counts and the top 100 were selected for further analysis. Parameters including citation density, publication year, journal, journal impact factor (IF), country, institution, author, study design, study field, evidence level, laser type, and keywords were meticulously analyzed. Results: The mean and standard deviations of total citation and citation density were 106.47 ± 65.76 and 7.61 ± 5.13, respectively. Positive and negative correlations were found between the number of citations and citation density and age of publication. While the mean number of citations was significantly higher in the period 2001–2010 compared to the other periods (*P* < 0.05), values were similar between the periods 1990–2000 and 2011–2014 (*P* > 0.05). Articles were mainly published in the *Journal of Endodontics*. The most productive country, institutions, and author were the United States, the University of Showa, and Koukichi Matsumoto. Diode and Er: YAG lasers were commonly investigated. Ex vivo studies were mainly performed followed by in vitro ones. The main study field was “antimicrobial effect”. Among keywords, “photodynamic therapy” was used more frequently. Conclusion: Lasers are predominantly utilized to leverage their antimicrobial efficacy. Advancements in technology will lead to improvements in the properties of lasers, thereby enhancing the disinfection of the root canal system.

**Supplementary Information:**

The online version contains supplementary material available at 10.1007/s10103-024-04163-3.

## Introduction

In modern dentistry, the integration of cutting-edge technologies has revolutionized traditional practices, offering more efficient and precise treatments. Regarding these innovations, lasers have emerged as a versatile tool with several applications, particularly in endodontics [1]. Weichman & Johnson pioneered the initial application of laser therapy in endodontics, employing a carbon dioxide (CO_2_) laser to seal the apical foramen [2]. Although they could not receive successful outcomes, it has pioneered future studies on the utility of lasers in various applications. Nowadays, lasers are used in endodontics, mainly for root canal irrigation activation along with shaping and canal filling, vital pulp therapies, pain management, and treatment of dentinal hypersensitivity [3–6]. In the last two decades, laser-activatedirrigation (LAI) has become increasingly popular for its effectiveness in enhancing disinfection in complex root canal structures containing microbial biofilms [7]. In furtherance, the position statement by the American Association of Endodontists (AAE), particularly highlighting its efficacy in disinfection compared to traditional methods, underscores the growing preference for LAI in modern endodontic practice [8].

Using lasers in different endodontic procedures has required the specification of appropriate laser types tailored to each treatment [9]. Commonly employed lasers in endodontics include erbium: yttrium–aluminum–garnet (Er: YAG), erbium, chromium-doped yttrium scandium gallium garnet (Er, Cr: YSGG), neodymium-doped yttrium aluminum garnet (Nd: YAG), neodymium: yttrium–aluminum–perovskite (Nd: YAP), CO_2_, and diode lasers [9, 10]. Er: YAG and Er, Cr: YSGG lasers are widely employed for endodontic irrigation, primarily for activating the irrigant. This is because their wavelengths (2,780 nm Er, Cr: YSGG, and 2,940 nm Er: YAG) are highly absorbed by water, generating a cavitation phenomenon within the fluid [11, 12]. Moreover, diode lasers are also preferred for disinfection purposes due to in vitro studies reporting their high antimicrobial efficacy on microorganisms isolated from the root canal system [13, 14]. Furthermore, according to clinical studies reporting the alleviation of postoperative pain following root canal treatment and retreatment, diode lasers can be utilized as a pain reduction strategy [4, 15, 16].

Bibliometric analysis represents a scientific review methodology supported by computer assistance, aiming to discern authors, institutions, or countries and their interconnections by comprehensively analyzing all academic publications pertinent to a specific topic or discipline [17]. The bibliometric analysis is mainly based on citation information describing the impact of publication and intellectual flow by presenting the obtained citation count over the years [18]. Scientometric analysis is a methodology employed to delve into bibliometric data, with the primary goal of gaining insights through visual representations of information related to keywords, authors, and countries [19]. This analysis allows readers to analyze the given topics’ complexity within an approach for visualization and mapping the knowledge area [19].

Although the bibliometric analysis of the application of lasers in dentistry has been conducted before [20], the analysis of the overall usage of lasers in endodontics, to the best of our knowledge, has not been performed yet. Since laser technology is also utilized in innovative areas, such as retrieving broken instruments or removing fiber posts along with conventional endodontic procedures, it is needed to map endodontic uses, categorize research, provide an overview of the scientific profile and inspire further research. Thus, this bibliometric and scientometric analysis aimed to identify and analyze the top most-cited 100 articles on the application of lasers in endodontics from 1990 to 2024.

## Materials and methods

A thorough electronic search was conducted using the “Clarivate Analytics Web of Science (WoS), All databases” (http://www.webofknowledge.com) as WoS is renowned for encompassing peer-reviewed, reputable scientific journals from across the globe. Moreover, WoS is an easy and adequate tool for bibliometric analysis and has been used in many studies effectively [21]. The related terms and search strategy were devised by three researchers (SNU, CK, and PB) possessing expertise in endodontics and/or bibliometrics.

Following the below-mentioned search queries were composed, the WoS was accessed for obtaining the articles with bibliometric data on February 15, 2024:

Q1: (((((((((((((((((((((((((((((((ALL=(laser*)) OR ALL=(laser therapy)) OR ALL=(low level laser therapies)) OR ALL=(low power laser therapies)) OR ALL=(high power laser*)) OR ALL=(laser dentistry)) OR ALL=(carbon dioxide)) OR ALL=(argon)) OR ALL=(erbium-doped yttrium aluminum garnet laser)) OR ALL=(Er: YAG)) OR ALL=(neodymium-doped yttrium aluminum garnet)) OR ALL=(Nd: YAG)) OR ALL=(erbium, chromium: yttrium-scandium gallium garne)) OR ALL=(Er, Cr: YSGG)) OR ALL=(diode laser)) OR ALL=(photodynamic therapy)) OR ALL=(antibacterial photodynamic therapy)) OR ALL=(antimicrobial photodynamic therapy)) OR ALL=(photoactivated disinfection)) OR ALL=(potassium titanyl phosphate)) OR ALL=(photobiomodulation therapy)) OR ALL=(laser induced photobiomodulation therapy)) OR ALL=(PIPS)) OR ALL=(SWEEPS)) OR ALL=(laser activated irrigation)) OR ALL=(laser assisted disinfection)) OR ALL=(laser assisted root canal treatment)) OR ALL=(laser assisted endodontics)) OR ALL=(KTP)) OR ALL=(ND: YAP)) OR ALL=(shock wave enhanced emission photoacoustic streaming)) OR ALL=(photon-induced photoacoustic streaming). 1,787,268 articles received.

Q2: (ALL = (endod*)) OR ALL = (root canal*). 77,640 articles received.

The two queries were combined as “Q1 AND Q2”, resulting in a total of 3915 articles. This bibliometric and scientometric investigation exclusively encompassed articles authored in English within the timeframe of 01.01.1990 to 01.01.2024 given the marked surge in the utilization of lasers in endodontics and associated advancements post-1990. Articles unrelated to the application of lasers in endodontics, conference papers, and editorial letters were excluded from the study’s purview.

**Fig. 1:**
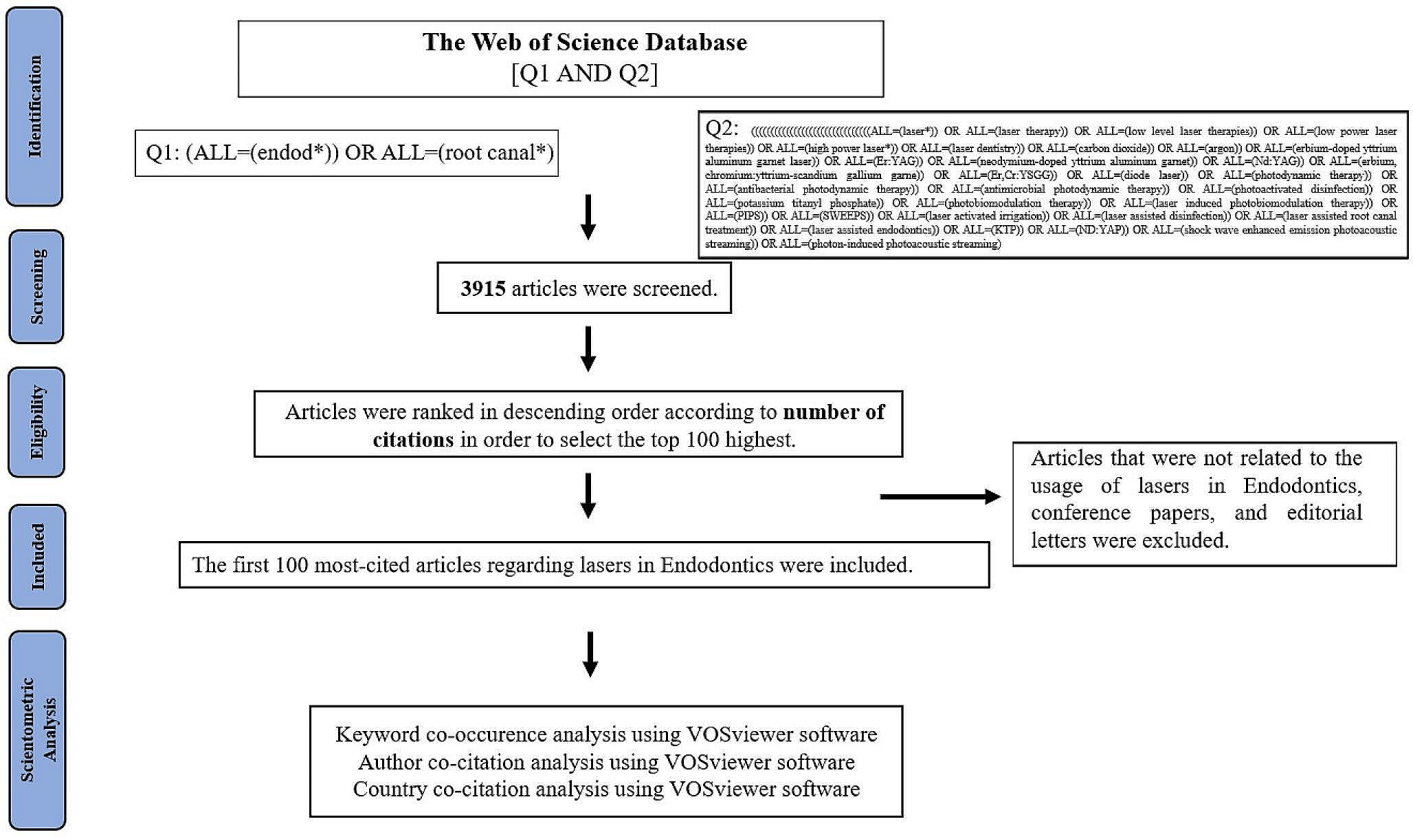
Searching process

Obtained bibliometric data from WoS was downloaded as a Microsoft Excel 15.0 (Microsoft, Redmond, WA, USA) file. The articles were ranked in descending order based on their citation counts. In cases where two articles have the same number of citations, the article with the higher citation density was ranked first. Two independent researchers (AC and SNU) conducted screening and analysis of the titles and/or abstracts to verify that the primary topic pertains to the utilization of lasers in endodontics. In cases where adequate information could not be obtained from the abstract and/or title, researchers accessed the full text. To ensure accuracy, any inconsistencies were addressed through consultation with the third and fourth researchers (CK and PB) until mutual agreement was reached. The search methodology is detailed in Fig. 1.

For each selected article, the following domains were recorded: citation count, citation density, publication year, age of publication, journal, impact factor (IF) sourced from Journal Citation Reports by Clarivate Analytics (2022), country of origin, affiliated institution, first author, co-authors, study field, study design, evidence level (EL), and keywords. Moreover, the used laser types in the studies were also extracted. The citation density was computed by dividing the total number of citations by the age of publication. Additionally, the included years were segmented into three decades (1990–2000, 2001–2010, and 2011–2024) to identify temporal changes in metrics. Study fields were classified collectively by all investigators, considering the prevalent focus areas evident in the selected articles. In addition, articles were classified according to the study types as follows: review (narrative, systematic review, and systematic review and meta-analysis), in vitro (a part of tooth, dentin slices, dentin powder, cell cultures, artificial tooth models), ex vivo (complete tooth), in vivo (animal studies), clinical observational study (case report), and clinical experimental study (randomized clinical trial (RCT), clinical trial). The preferred laser types, the usage fields, and main findings were also examined.

Following the bibliometric analysis, scientometric analysis was undertaken to systematically map the scientific knowledge domain, delineate research themes, and elucidate associated challenges. The selected articles underwent further scrutiny regarding the geographical distribution of the most contributing authors. Furthermore, both domestic and international collaborations among authors and countries were identified and visually represented. Additionally, keywords were analyzed and depicted visually.

To synthesize and visually represent the bibliometric data gathered from WoS and construct a scientific map, we employed the VOSviewer (version 1.6.18; Leiden University Center for Science and Technology Studies, Leiden, The Netherlands, accessible at https://www.vosviewer.com). A minimum threshold of one document per author and country, and zero citations, was set to optimize the analysis. In this visualization, node size corresponds to the frequency of analyzed parameters; hence, larger nodes indicate higher frequencies. Furthermore, the thickness of edges indicates the strength of interactions between nodes, with their colors denoting the respective keyword clusters [18].

All statistical analyses were performed using the Statistical Package for Social Sciences (SPSS) for Windows software, version 26.0 (SPSS Inc., Chicago, IL, USA). The Shapiro-Wilk test was employed to assess the normality of both the number of citations and citation density. The Kruskal-Wallis test was employed to compare these metrics among decades. In cases where the results were statistically significant, pair-by-pair comparisons were made using the Mann-Whitney U test. The correlation between citation count, citation density, age of publication, journal’s IF, and number of citations was evaluated using the square of the Spearman linear coefficient. The significance level was set at *P* < 0.05.

## Results

The most-cited 100 articles regarding the usage of lasers in endodontics received 10,647 citations in total while the total citation density count was 761.20. The mean and standard deviations of total citation and citation density were 106.47 ± 65.76 and 7.61 ± 5.13, respectively. The most-cited 100 articles are presented in Supplementary Material Table 1, along with citation-based information and main findings.

**Table 1:** The comparison of the number of citations and citation density values of the top 100 most-cited articles about lasers in endodontics regarding decades

	1990–2000 *n* = 22	2001–2010 *n* = 42	2011–2024 *n* = 36	Comparison *p* value*
**Number of citations** ^**a**^	86.72^1^	122.90^2^	99.36^1^	< 0.05
Min - Max	59– 165	59– 493	59–417	
Total	1908	5162	3577	
**Citation density** ^**a**^	4.11^1^	7.23^2^	10.20^3^	< 0.05
Min-Max	2.17–15.25	3.11–27.39	1.97–32.08	
Total	90.36	303.76	367.08	

^a^ Mean value. Min - Max: Minimum and Maximum values

* The Shapiro-Wilk test showed no normality. The Mann-Whitney U test analysed pair comparisons. Read horizontally; the different superscript numbers indicate a significant difference

**Fig. 2:**
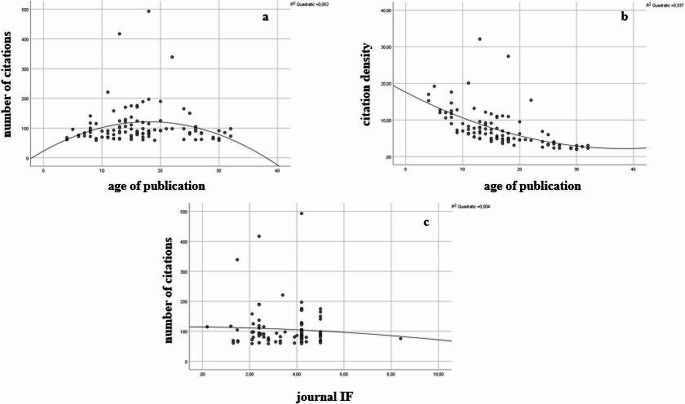
Associations between the number of citations and age of publication (**a**), the citation density and age of publication (**b**), journal IF and number of citations (**c**)

A positive correlation between citation and age of publication was found without a significant difference (correlation coefficient = 0.028, *P* > 0.05, R^2^: 0.062). However, there was a statistically significant negative correlation between citation density and age of publication (correlation coefficient = -0.759, *P* < 0.001, R^2^: 0.337). Moreover, there was a positive correlation between citation and IF without a significant difference (correlation coefficient = 0.028, *P* > 0.05, R^2^: 0.004). The associations are shown in Fig. 2.

Among 3 decades, both the number of citations and citation density values differed significantly (*P* < 0.05). While the mean number of citations was significantly higher in the time period 2001–2010 compared to the other periods (*P* < 0.05), values were similar between the periods 1990–2000 and 2011–2014 (*P* > 0.05). Regarding citation density, significantly higher and lower values were observed in 2011–2024 and 1990–2000, respectively. Table 1 presents metrics for the top 100 most-cited articles about lasers in endodontics in periods. Accordingly, the highest number of articles were published between the time period 2001–2010 (*n* = 42) and in the years 2006 (*n* = 8) and 2011 (*n* = 8) followed by 2007, 2008, and 2009 (*n* = 7 for each).

### Journal and IF

Twenty-five different journals contributed to this bibliometric and scientometric study. Among them, the *Journal of Endodontics* had the highest number of publications (*n* = 34), followed by the *International Endodontic Journal* (*n* = 15), and Lasers in Surgery and Medicine (*n* = 12). According to the Journal Citation Reports in 2022, the 5 journals with the highest IFs were the *Cochrane Database of Systematic Reviews* (*n* = 1, IF:8.4), *International Endodontic Journal* (*n* = 15, IF:5), *Journal of Dentistry* (*n* = 2, IF: 4.4), *Journal of Endodontics* (*n* = 34, IF: 4.2), and *Frontiers in Physiology* (*n* = 1, IF:4).

### Countries, institutions, and authors

A total of 25 countries participated in this study. The United States of America (US) was the most productive country with 24 publications, followed by Brazil (*n* = 9). Accordingly, co-authorship analysis of the countries revealed that the US collaborated with the highest number of countries (*n* = 7). The majority of the countries were associated with domestic collaborations. Moreover, the first 3 countries that had the highest total link strength were the US (*n* = 12), Brazil (*n* = 6), and the United Kingdom (*n* = 4).

Sixty-three institutions were retrieved according to the affiliation of the first author. The University of Showa made the highest scientific contribution (*n* = 7), followed by the University of Harvard (*n* = 6) and the University of Vienna (*n* = 6). The first ten most contributed countries and the institutions with at least 2 publications are given in Table 2.

**Table 2:** The countries and institutions with at least 2 publications

Countries	Number of Publications
United States of America	24
Brazil	9
Japan	8
Belgium	7
Austria	6
United Kingdom	5
China	4
Germany	4
Iran	4
Türkiye	4
**Institutions**	
University of Showa	7
University of Harvard	6
University of Vienna	6
University of Ghent	5
Centro de Laser e Aplicações	4
Private Dental Practice	3
Academic Center for Dentistry Amsterdam	3
University of Aachen	2
University of Queensland	2
The Forsyth Institute	2
University of North Carolina at Chapel Hill	2
University of Leuven	2
University of Pekin	2
Queen Mary University of London	2
University of Tehran	2
University of Sao Paulo	2
University of Illinois	2

In total, 480 authors contributed to the list of the top 100 most-cited articles about lasers in endodontics. Koukichi Matsumoto was the most productive author with 8 publications, followed by Andreas Moritz and Yuichi Kimura with 7 articles. Tom C. Pagonis, Kawe Goharkhay, Carla R. Fontana, Roeland J.G. De Moor, Michael R. Hamblin, Maarten Meire, Ulrich Schoop, and Wolfgang Sperr had 6 publications. However, Aguinaldo Silva Garcez (*n* = 5) was the most-seen first author, followed by Frank H. Takeda (*n* = 4). Regarding collaborations, Nikolaos S. Soukos (*n* = 62) and Tom C. Pagonis (*n* = 54) had the highest total link strength.

**Fig. 3:**
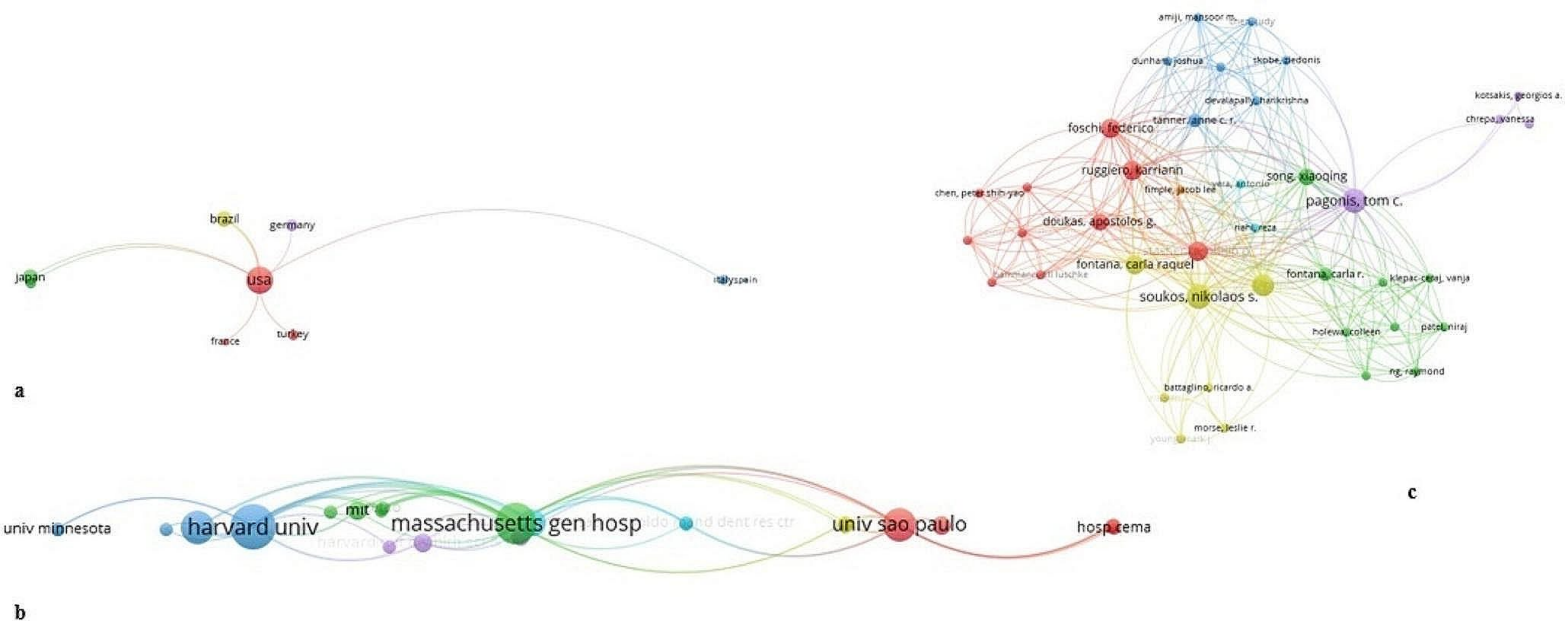
International collaborations between participated countries (**a**), institutions (**b**), and authors (**c**)

International collaborations between participating countries, institutions, and authors are presented in Fig. 3.

### Study type study field, EL, laser types and keywords

Ex vivo (*n* = 39) studies were mainly performed followed by in vitro (*n* = 30) studies and reviews (*n* = 19). Thirteen study fields were determined by 3 investigators (SNU, CK, and PB). “Antimicrobial effect” was the dominant research area with 62 publications. Study types and fields are demonstrated in Table 3. In addition, according to the EL pyramid [22], studies had mostly V (*n* = 88), I (*n* = 5), II (*n* = 5), and III (*n* = 2) levels.

**Table 3:** Study types and fields of the top 100 most-cited articles about lasers in endodontics

	*n*	In vitro	Ex vivo	Narrative Review	Systematic Review	Systematic Review and Meta-analysis	Animal Study	Case Report	Randomized Clinical Trial	Clinical Trial
Review	19	-	-	13	4	2	-	-	-	-
In vitro	30	30	-	-	-	-	-	-	-	-
Ex vivo	39	-	39	-	-	-	-	-	-	-
In vivo	4		3	-	-	-	1	-	-	-
Clinical observational study	1	-	-	-	-	-	-	1	-	-
Clinical experimental study	7	-	-	-	-	-	-	-	5	2
**Study Fieds**						**n**				
Endodontic surgery						1				
Antimicrobial effect						62				
Smear layer removal						13				
Debris removal						2				
Dentine hypersensitivity						4				
Diagnosis						6				
Review / General view						1				
Cavity preparation						1				
Bleaching						3				
Pain management						2				
Vital pulp therapy						1				
Biological aspect						2				
Disinfection effect						2				

Except for the review articles which include various laser types, the articles mainly investigated the specific laser types as follows: argon (Ar), argon floride (ArF), CO_2,_ diode, Er, Cr: YSGG, Er: YAG, gallium aluminum arsenide (GaAlAs), Helium-neon (He-Ne), potassium titanyl phosphate (KTP), Nd: YAG, and Nd: YAP. In addition, Laser Doppler Flowmetry was also used for diagnostic purposes. Among them, diode (*n* = 40) and Er: YAG (*n* = 31) lasers were the most investigated types.

**Fig. 4:**
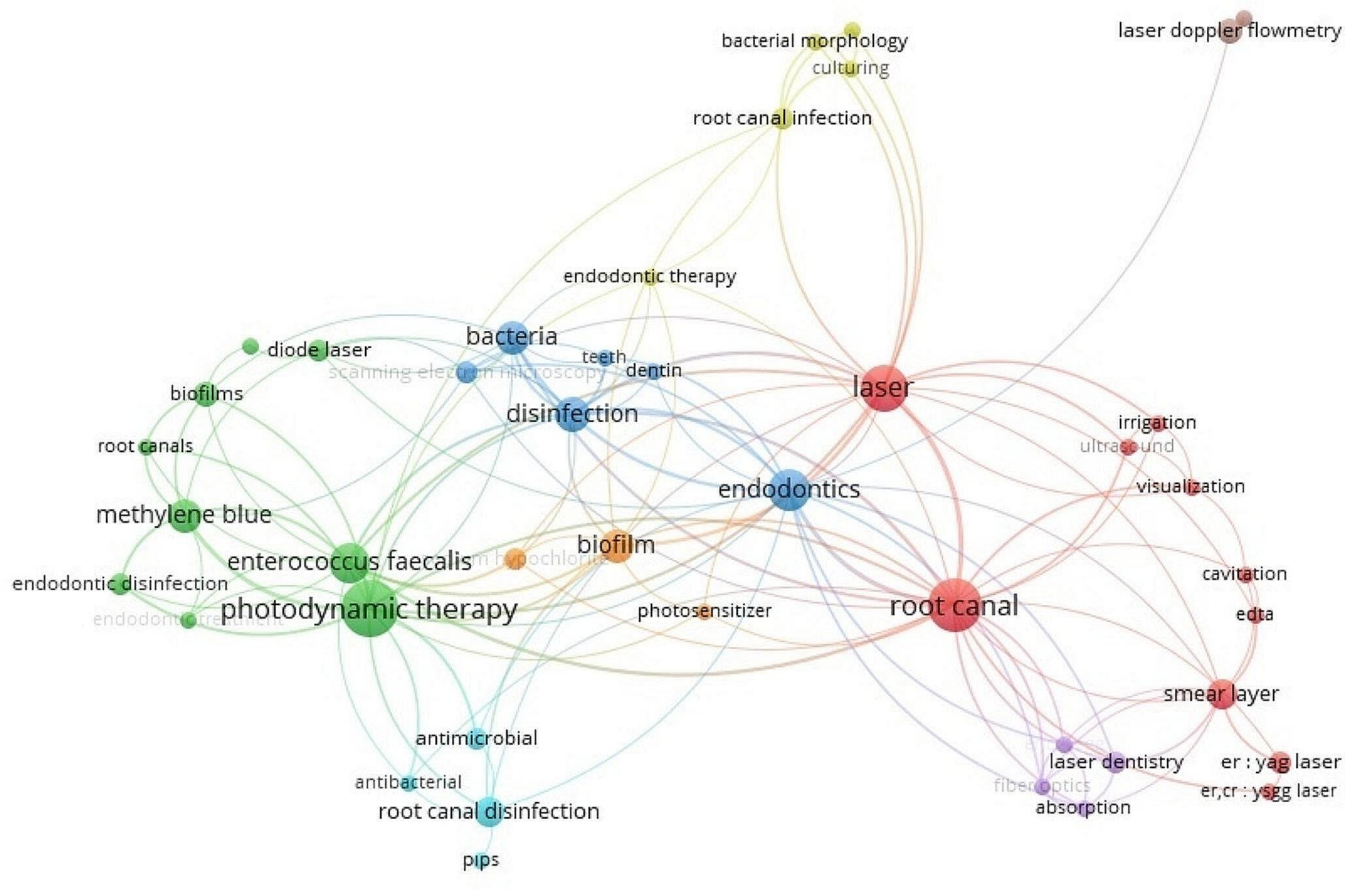
The visualization map of frequently used keywords

The VOSviewer obtained 197 keywords in total. To enhance the clarity, the co-occurrence was decreased to at least 2 articles. Consequently, 47 keywords (nodes) met the threshold, and these were grouped into 8 clusters, and a maximum of 1,000 lines were loaded. “Photodynamic therapy” (*n* = 21, total link strength = 42) was the most preferred keywords followed by “root canal” (*n* = 18, total link strength = 52) and “laser” (*n* = 14, total link strength = 41). Figure 4 presents the visualization map of keywords used in selected studies.

## Discussion

Research in the field of lasers in endodontics has been growing steadily, with numerous studies investigating the efficacy, safety, and clinical outcomes associated with various laser modalities. In this sense, bibliometric analysis can help researchers and dental practitioners better understand the landscape of this evolving field and contribute to the advancement of the usage of lasers in endodontic practice. Therefore, this bibliometric and scientometric study endeavors to demonstrate the prevailing global research patterns concerning lasers in endodontics using the citation analysis.

In accordance with the nature of citation analysis, a positive correlation between number of citations and age of publication was observed which supports the expectation that older articles tend to receive more citation then new ones [23]. However, the highest citation counts were obtained in the period 2001–2010. This can be attributed to the growing utilization of lasers in endodontic procedures coupled with notable advancements in technology during the 2000s and consequently publication of pioneer papers in this field. On the other hand, the citation density values were significantly higher in the period 2011–2024. While this suggests that articles published during this period received significant citations in a relatively short timeframe, it is crucial to scrutinize the long-term citation trends of these publications [24]. In particular, numerous studies are being conducted on new types of lasers and their applications in endodontics, however, it needs time to understand their scientific contributions to literature.

There was a positive moderate correlation between number of citations and journals IFs. This is consistent with papers in established journals in endodontics with strong reputations and high citation rates, such as the *Journal of Endodontics* and the *International Endodontic Journal*, attracting more citations than newer journals, or those covering non-specialized areas [25]. Additionally, there was a global spread of countries involved in the top 100 publications, with sizeable contributions from the US, the European Union, Brazil, and South-East Asia. This indicates that activity in research into laser applications in endodontics is distributed widely across the globe, despite variations in the extent of public funding for dental research between jurisdictions [21, 26].

The majority of the included studies were designed as ex vivo and in vitro for assessing the various features of lasers in endodontics. Both study designs contribute significantly to the advancement of endodontic practice by providing essential data on the efficacy, safety, and suitability of various treatment modalities. In addition, they serve as essential steps in the research and development process, helping to bridge the gap between basic science discoveries and clinical application in endodontics. However, in order to evaluate the clinical impacts and effects of lasers, well-designed RCTs are needed to be performed more frequently.

The main study field was “antimicrobial effect” that indicates the publications evaluated the effectiveness of lasers in microbiological aspect. Lasers are primarily employed in endodontics for disinfecting the root canal system, as conventional methods often pose a risk to achieve complete disinfection due to the intricate anatomical structures involved. This necessity for thorough disinfection has driven the development of new technologies, with lasers emerging as promising tools for addressing this challenge [27, 28]. Moreover, the most used keyword was “Photodynamic therapy”. Microorganisms, including bacteria, fungi, viruses, and protozoa, can be eradicated by singlet oxygen species generated through the reaction of sensitizers with specific wavelength [29]. Thus, photodynamic therapy concepts have been proposed to enhance root canal disinfection either by replacing traditional procedures or by supplementing their effects [30].

Various type of lasers have been studied in endodontics field in order to observe the mechanisms and effectiveness. In this bibliometric study, diode and Er: YAG lasers were the mostly preferred. Diode lasers, in particular, have demonstrated the ability to significantly improve root canal disinfection, leading to successful outcomes in vivo [31] and notably reducing post-operative pain [32]. Especially, it is important to mention that the antibacterial action of diode laser occurs through heat transmission or its interaction with external chromophores [33]. Similarly, Er: YAG lasers also can be used for removing smear layer and biofilms along with its dental hard and soft tissue ablation ability [34]. Nowadays, photon induced photoacoustic streaming (PIPS) and shock wave-enhanced emission photoacoustic streaming (SWEEPS) technologies have been introduced through the usage of Er: YAG lasers at low settings (20 mJ, 15 Hz) and ultra-short laser pulses (50 µs). It has been asserted that this energy is capable of generating cavitation phenomena and shockwaves, thereby enhancing irrigant agitation [35]. Accordingly, it is anticipated that future studies will focus on enhancing the effectiveness of laser types by modifying their properties.

It is important to acknowledge certain limitations within the scope of this bibliometric study. Firstly, the 100 most-cited articles were retrieved from a single database. While the WoS is widely regarded as a prestigious and suitable database for bibliometric analysis, utilizing only one database may introduce a potential source of bias. Additionally, the time-dependent nature of citation analysis may not fully capture the scientific merit of studies, making it challenging to assess the impact of recently published influential works. Moreover, self-citations could potentially introduce bias into the analysis, as authors may tend to promote the citation of their own studies.

## Conclusion

It is revealed that laser applications were primarily used to enhance the disinfection efficacy in endodontic treatments. In many studies spearheaded by the US government, diode and Er: YAG lasers have been commonly utilized. This bibliometric study provides a direction for future research by highlighting the existing knowledge and frontiers within laser applications in the endodontic field.

## Electronic supplementary material

Below is the link to the electronic supplementary material.

Supplementary Material 1
