# Supplementary material for: The cutting-edge roles of lasers in endodontics: A bibliometric and scientometric analysis of the 100 most-cited articles
Source: Lasers Med Sci. 2024 Aug 16;39(1):219. doi: 10.1007/s10103-024-04163-3 (PMC11327183; doi:10.1007/s10103-024-04163-3)
Supplement: Supplementary file 1 — Supplementary Material 1 [file 10103_2024_4163_MOESM1_ESM.docx]

**Supplementary Material Table 1.** The 100 most-cited articles about lasers in endodontics

| **Citation**  **Rank** | **Reference** | **No. Citation** | **No. Citation density (rank)** | **Main Findings** |
| --- | --- | --- | --- | --- |
| 1 | Kim S, Kratchman S. Modern endodontic surgery concepts and practice: a review. J Endod. 2006; 32: 601-23. | 493 | 27.39 (2) | The CO2 or Nd/Yag lasers are not suitable because they do not cut bone and dentin effectively. Er:Yag lasers have also been used for apical surgery. |
| 2 | Kharkwal GB, Sharma SK, Huang YY, Dai T, Hamblin MR. Photodynamic therapy for infections: clinical applications. Lasers Surg Med. 2011; 43: 755-67. | 417 | 32.08 (1) | As yet clinical PDT for infections has been mainly in the field of dermatology using 5-aminolevulanic acid and in dentistry using phenothiazinium dyes. We expect more to see applications of PDT to more challenging infections using advanced antimicrobial photosensitizers targeted to microbial cells in the years to come. |
| 3 | Torabinejad M, Handysides R, Khademi AA, Bakland LK. Clinical implications of the smear layer in endodontics: a review. Oral Surg Oral Med Oral Pathol Oral Radiol Endod. 2002; 94: 658-66. | 339 | 15.41 (7) | Lasers are effective in smear layer removal. |
| 4 | Gursoy H, Ozcakir-Tomruk C, Tanalp J, Yilmaz S. Photodynamic therapy in dentistry: a literature review. Clin Oral Investig. 2013; 17: 1113-25. | 221 | 20.09 (3) | PDT seems to be an effective tool in the treatment of localized and superficial infections. Within the limitations of the present review, it can be concluded that although PDT cannot replace antimicrobial therapy at its current stage, it may be used as an adjunctive tool for facilitating the treatment of oral infections. |
| 5 | Soukos NS, Chen PS, Morris JT, et al. Photodynamic therapy for endodontic disinfection. J Endod. 2006; 32: 979-84. | 197 | 10.94 (21) | It was concluded that PDT may be developed as an adjunctive procedure to kill residual bacteria in the root canal system after standard endodontic treatment. |
| 6 | Schoop U, Kluger W, Moritz A, et al. Bactericidal effect of different laser systems in the deep layers of dentin. Lasers Surg Med. 2004; 35: 111-6. | 190 | 9.50 (26) | The present study demonstrates that all the wavelengths investigated are suitable for the disinfection of even the deeper layers of dentin and may prove to constitute valuable tools in state-of-the-art endodontics. |
| 7 | Garcez AS, Ribeiro MS, Tegos GP, et al. Antimicrobial photodynamic therapy combined with conventional endodontic treatment to eliminate root canal biofilm infection. Lasers Surg Med. 2007; 39: 59-66. | 189 | 11.12 (19) | Bioluminescence imaging is an efficient way to monitor endodontic therapy. Antimicrobial PDT may have a role to play in optimized endodontic therapy. |
| 8 | Garcez AS, Nuñez SC, Hamblin MR, Ribeiro MS. Antimicrobial effects of photodynamic therapy on patients with necrotic pulps and periapical lesion. J Endod. 2008; 34: 138-42. | 176 | 11.00 (20) | Results suggest that the use of PDT added to endodontic treatment leads to an enhanced decrease of bacterial load and may be an appropriate approach for the treatment of oral infections. |
| 9 | de Groot SD, Verhaagen B, Versluis M, et al. Laser-activated irrigation within root canals: cleaning efficacy and flow visualization. Int Endod J. 2009; 42: 1077-83. | 175 | 11.67 (18) | The in vitro recordings suggest that streaming, caused by the collapse of the laser-induced bubble, is the main cleaning mechanism of LAI. |
| 10 | Fimple JL, Fontana CR, Foschi F, et al. Photodynamic treatment of endodontic polymicrobial infection in vitro. J Endod. 2008; 34: 728-34. | 171 | 10.69 (23) | PDT achieved up to 80% reduction of colony-forming unit counts. PDT can be an effective adjunct to standard endodontic antimicrobial treatment when the PDT parameters are optimized. |
| 11 | Pagonis TC, Chen J, Fontana CR, et al. Nanoparticle-based endodontic antimicrobial photodynamic therapy. J Endod. 2010; 36: 322-8. | 170 | 12.14 (14) | The utilization of PLGA nanoparticles encapsulated with photoactive drugs may be a promising adjunct in antimicrobial endodontic treatment. |
| 12 | Kimura Y, Wilder-Smith P, Matsumoto K. Lasers in endodontics: a review. Int Endod J. 2000; 33: 173-85. | 165 | 6.88 (43) | The role of lasers in endodontics since the early 1970s, summarizes many research reports from the last decade, and surmises what the future may hold for lasers in endodontics. With the potential availability of many new laser wavelengths and modes, much interest is developing in this promising field. |
| 13 | DiVito E, Peters OA, Olivi G. Effectiveness of the erbium:YAG laser and new design radial and stripped tips in removing the smear layer after root canal instrumentation. Lasers Med Sci. 2012; 27: 273-80. | 158 | 13.17 (10) | The study showed that standardized instrumentation, followed by a final Er:YAG laser irradiation in wet canals with EDTA irrigation resulted in more cleaning of the root canal walls and a higher quantity of open tubules in comparison with the traditional irrigation method. |
| 14 | Takeda FH, Harashima T, Kimura Y, Matsumoto K. A comparative study of the removal of smear layer by three endodontic irrigants and two types of laser. Int Endod J. 1999; 32: 32-9. | 150 | 6.00 (54) | The CO2 laser was useful in removing and melting the smear layer on the instrumented root-canal walls and the Er:YAG laser was the most effective in removing the smear layer from the root-canal wall. |
| 15 | Neelakantan P, Cheng CQ, Mohanraj R, et al. Antibiofilm activity of three irrigation protocols activated by ultrasonic, diode laser or Er:YAG laser in vitro. Int Endod J. 2015; 48: 602-10. | 141 | 17.63 (5) | Diode laser and Er:YAG laser activation were superior to ultrasonics in dentinal tubule disinfection. |
| 16 | Blanken J, De Moor RJ, Meire M, Verdaasdonk R. Laser induced explosive vapor and cavitation resulting in effective irrigation of the root canal. Part 1: a visualization study. Lasers Surg Med. 2009; 41: 514-9. | 137 | 9.13 (28) | Imaging suggests that the working mechanism of an Er,Cr:YSGG laser in root canal treatment in an irrigation solution can be attributed to cavitation effects inducing high-speed fluid motion into and out the canal. |
| 17 | Garcez AS, Nuñez SC, Hamblim MR, Suzuki H, Ribeiro MS. Photodynamic therapy associated with conventional endodontic treatment in patients with antibiotic-resistant microflora: a preliminary report. J Endod. 2010; 36: 1463-6. | 129 | 9.21 (27) | The use of PDT added to conventional endodontic treatment leads to a further major reduction of microbial load. PDT is an efficient treatment to kill multi-drug resistant microorganisms. |
| 18 | Gutknecht N, Franzen R, Schippers M, Lampert F. Bactericidal effect of a 980-nm diode laser in the root canal wall dentin of bovine teeth. J Clin Laser Med Surg. 2004; 22: 9-13. | 125 | 6.25 (51) | The results of this research show that the 980-nm diode laser can eliminate bacteria that have immigrated deep into the dentin, thus being able to increase the success rate in endodontic therapy. |
| 19 | Peters OA, Bardsley S, Fong J, Pandher G, Divito E. Disinfection of root canals with photon-initiated photoacoustic streaming. J Endod. 2011; 37: 1008-12. | 124 | 9.54 (25) | Activated disinfection did not completely remove bacteria from the apical root canal third and infected dentinal tubules. However, the fact that laser activation generated more negative bacterial samples and left less apical bacteria/biofilm than ultrasonic activation warrants further investigation. |
| 20 | George R, Meyers IA, Walsh LJ. Laser activation of endodontic irrigants with improved conical laser fiber tips for removing smear layer in the apical third of the root canal. J Endod. 2008; 34: 1524-7. | 124 | 7.75 (35) | Lasing improved the action of ethylene diamine tetraacetic acid with cetavlon (EDTAC) in removing smear layer. Conical fibers performed better than plain fibers, but there was no difference in performance between the 2 laser systems when matched for all other parameters. |
| 21 | Souza LC, Brito PR, de Oliveira JC, et al. Photodynamic therapy with two different photosensitizers as a supplement to instrumentation/irrigation procedures in promoting intracanal reduction of Enterococcus faecalis. J Endod. 2010; 36: 292-6. | 122 | 8.71 (31) | These in vitro results suggest that PDT with either MB or TB may not exert a significant supplemental effect to instrumentation/irrigation procedures with regard to intracanal disinfection. |
| 22 | Foschi F, Fontana CR, Ruggiero K, et al. Photodynamic inactivation of Enterococcus faecalis in dental root canals in vitro. Lasers Surg Med. 2007; 39: 782-7. | 119 | 7.00 (41) | PDT achieved 77.5% reduction of E. faecalis viability. MB alone and light alone reduced bacterial viability by 19.5% and 40.5%, respectively. HPLC did not reveal any porphyrin patterns expressed by E. faecalis. |
| 23 | Carrera ET, Dias HB, Corbi SCT, et al. The application of antimicrobial photodynamic therapy (aPDT) in dentistry: a critical review. Laser Phys. 2016; 26. | 117 | 14.63 (9) | The goal of this review was to summarize the results of research on aPDT in dentistry using the PubMed database focusing on recent studies of the effectiveness aPDT in decreasing microorganisms and microbial biofilms, and also to describe aPDT effects, mechanisms of action and applications. |
| 24 | Pruthi PJ, Yadav N, Nawal RR, Talwar S, Lamba AK. Novel Use of PRF and PDT in the Management of Trauma Induced Root Resorption and Infrabony Defect. J Clin Diagn Res. 2015; 9: Zd26-8. | 115 | 12.78 (11) | To allow faster regeneration of the periodontal tissues, Platelet rich fibrin (PRF), a second generation platelet concentrate was used as an apical matrix over which MTA plug was given. The periodontal defect was managed with the help of localized antimicrobial photodynamic therapy (aPDT). |
| 25 | Bonsor SJ, Nichol R, Reid TM, Pearson GJ. Microbiological evaluation of photo-activated disinfection in endodontics (an in vivo study). Br Dent J. 2006; 200: 337-41, discussion 29. | 115 | 6.39 (49) | The PAD system offers a means of destroying bacteria remaining after using conventional irrigants in endodontic therapy. |
| 26 | De Moor RJ, Blanken J, Meire M, Verdaasdonk R. Laser induced explosive vapor and cavitation resulting in effective irrigation of the root canal. Part 2: evaluation of the efficacy. Lasers Surg Med. 2009; 41: 520-3. | 111 | 7.40 (39) | Under the conditions of this study LAI is statistically significantly more effective in removing artificially placed dentin debris in a root canal as PUI and CI. |
| 27 | De Moor RJ, Meire M, Goharkhay K, Moritz A, Vanobbergen J. Efficacy of ultrasonic versus laser-activated irrigation to remove artificially placed dentin debris plugs. J Endod. 2010; 36: 1580-3. | 106 | 7.57 (37) | LAI techniques using erbium lasers (Er:YAG or Er,Cr:YSGG) for 20 seconds (4x 5 seconds) are as efficient as PUI with the intermittent flush technique (3x 20 seconds). |
| 28 | Rizoiu I, Kohanghadosh F, Kimmel AI, Eversole LR. Pulpal thermal responses to an erbium,chromium: YSGG pulsed laser hydrokinetic system. Oral Surg Oral Med Oral Pathol Oral Radiol Endod. 1998; 86: 220-3. | 105 | 4.04 (80) | The erbium,chromium:yttrium-scandium-gallium-garnet laser-powered hydrokinetic system, when used for cavity preparation, had no apparent adverse thermal effect as measured in the pulp space. |
| 29 | Rios A, He J, Glickman GN, et al. Evaluation of photodynamic therapy using a light-emitting diode lamp against Enterococcus faecalis in extracted human teeth. J Endod. 2011; 37: 856-9. | 104 | 8.00 (33) | PDT using TBO and a LED lamp has the potential to be used as an adjunctive antimicrobial procedure in conventional endodontic therapy. |
| 30 | Matsumoto H, Yoshimine Y, Akamine A. Visualization of irrigant flow and cavitation induced by Er:YAG laser within a root canal model. J Endod. 2011; 37: 839-43. | 101 | 7.77 (34) | These results suggest that the cleaning mechanism of an Er:YAG laser within the root canal might depend on rapid fluid motion caused by expansion and implosion of laser-induced bubbles. |
| 31 | Azim AA, Aksel H, Zhuang T, et al. Efficacy of 4 Irrigation Protocols in Killing Bacteria Colonized in Dentinal Tubules Examined by a Novel Confocal Laser Scanning Microscope Analysis. J Endod. 2016; 42: 928-34. | 100 | 12.50 (13) | XP Endo appears to be more efficient than other 3 techniques in disinfecting the main canal space and up to 50 μm deep into the dentinal tubules. PIPS appears to be most effective in killing the bacteria deep in the dentinal tubules. |
| 35 | George S, Kishen A. Photophysical, photochemical, and photobiological characterization of methylene blue formulations for light-activated root canal disinfection. J Biomed Opt. 2007; 12: 034029. | 98 | 5.76 (55) | The efficacy of LAT is evaluated on biofilms produced by both organisms under in vitro and ex vivo conditions. A dual-stage approach that applies a photosensitization medium and an irradiation medium separately is tested. The MIX-based photosensitization medium in combination with dual-stage approach demonstrates thorough disinfection of the root canal with bacterial biofilms. This method will have potential application for root canal disinfection. |
| 32 | Kashima-Tanaka M, Tsujimoto Y, Kawamoto K, et al. Generation of free radicals and/or active oxygen by light or laser irradiation of hydrogen peroxide or sodium hypochlorite. J Endod. 2003; 29: 141-3. | 98 | 4.67 (70) | The amounts of hydroxyl radical generated from H2O2 after irradiation were in the order: plasma lamp > halogen lamp > He-Ne laser > Yellow He-Ne laser. On the other hand, the amounts of DMPO-X generated from NaClO after irradiation were in the order: plasma lamp > Yellow He-Ne laser > halogen lamp > He-Ne laser. |
| 36 | Seal GJ, Ng YL, Spratt D, Bhatti M, Gulabivala K. An in vitro comparison of the bactericidal efficacy of lethal photosensitization or sodium hyphochlorite irrigation on Streptococcus intermedius biofilms in root canals. Int Endod J. 2002; 35: 268-74. | 98 | 4.45 (75) | The combined use of a photosensitizing agent and a low power laser directed at the access cavity was bactericidal to S. intermedius biofilms in root canals but was unable to achieve total kill, unlike 3% NaOCl. |
| 37 | Gutknecht N, van Gogswaardt D, Conrads G, et al. Diode laser radiation and its bactericidal effect in root canal wall dentin. J Clin Laser Med Surg. 2000; 18: 57-60. | 98 | 4.08 (79) | This investigation indicates that the diode laser radiation reduces the number of bacteria in deep layers of infected root canal wall dentin. |
| 33 | Takeda FH, Harashima T, Kimura Y, Matsumoto K. Comparative study about the removal of smear layer by three types of laser devices. J Clin Laser Med Surg. 1998; 16: 117-22. | 98 | 3.77 (82) | These results show that the argon laser and Nd:YAG laser are useful to remove the smear layer and that the Er:YAG laser irradiation is the most effective to remove the smear layer on root canal walls. |
| 34 | K Levy G. Cleaning and shaping the root canal with a Nd:YAG laser beam: a comparative study. J Endod. 1992; 18: 123-7. | 98 | 3.06 (89) | Scanning electron microscopic evaluations showed that preparation with a laser beam is possible and results in an improvement in the cleanliness of the canal walls when compared with conventional techniques. |
| 38 | Chen E, Abbott PV. Dental pulp testing: a review. Int J Dent. 2009; 2009: 365785. | 97 | 6.47 (47) | Whilst the prospect is promising, there are still many practical issues that need to be addressed before vitality tests can replace sensibility tests as the standard clinical pulp diagnostic test. With all pulp tests, the results need to be carefully interpreted and closely scrutinised as false results can lead to misdiagnosis which can then lead to incorrect, inappropriate, or unnecessary treatment. |
| 39 | Plotino G, Grande NM, Mercade M. Photodynamic therapy in endodontics. Int Endod J. 2019; 52: 760-74. | 96 | 19.20 (4) | Current publications tested PDT in terms of bacterial load reduction in vivo, in vitro and ex vivo, showing promising results. |
| 40 | Akcay M, Arslan H, Durmus N, Mese M, Capar ID. Dentinal tubule penetration of AH Plus, iRoot SP, MTA fillapex, and guttaflow bioseal root canal sealers after different final irrigation procedures: A confocal microscopic study. Lasers Surg Med. 2016; 48: 70-6. | 94 | 11.75 (17) | The dentinal tubule penetration area was significantly affected by the selection of root canal sealer, final irrigation procedure, and root canal third. Use of iRoot with PIPS tip or PUI seems advantageous in dentinal tubule penetration. |
| 41 | Eldeniz AU, Usumez A, Usumez S, Ozturk N. Pulpal temperature rise during light-activated bleaching. J Biomed Mater Res B Appl Biomater. 2005; 72: 254-9. | 94 | 4.95 (64) | Light activation of bleaching materials with diode laser caused higher temperature changes as compared to other curing units and the temperature rise detected was viewed as critical for pulpal health. |
| 42 | Cheng X, Guan S, Lu H, et al. Evaluation of the bactericidal effect of Nd:YAG, Er:YAG, Er,Cr:YSGG laser radiation, and antimicrobial photodynamic therapy (aPDT) in experimentally infected root canals. Lasers Surg Med. 2012; 44: 824-31. | 93 | 7.75 (36) | All the laser radiation protocols tested, especially Er:YAG/NaClO/NS/DW, have effective bactericidal effect in experimentally infected root canals. Er:YAG/NaClO/NS/DW seems to be an ideal protocol for root canal disinfection during endodontic therapy. |
| 43 | Kreisler MB, Haj HA, Noroozi N, Willershausen B. Efficacy of low level laser therapy in reducing postoperative pain after endodontic surgery-- a randomized double blind clinical study. Int J Oral Maxillofac Surg. 2004; 33: 38-41. | 92 | 4.60 (71) | Low level laser therapy can be beneficial for the reduction of postoperative pain. Its clinical efficiency and applicability with regard to endodontic surgery, however require further investigation. This is in particular true for the optimal energy dosage and the number of laser treatments needed after surgery. |
| 47 | Bago I, Plečko V, Gabrić Pandurić D, et al. Antimicrobial efficacy of a high-power diode laser, photo-activated disinfection, conventional and sonic activated irrigation during root canal treatment. Int Endod J. 2013; 46: 339-47. | 91 | 8.27 (32) | The PAD and EndoActivator system were more successful in reducing the root canal infection than the diode laser and NaOCl syringe irrigation alone. |
| 44 | Schoop U, Kluger W, Dervisbegovic S, et al. Innovative wavelengths in endodontic treatment. Lasers Surg Med. 2006; 38: 624-30. | 91 | 5.06 (60) | The present study demonstrates that both wavelengths investigated could be suitable for the disinfection of even the deeper layers of dentin and equal the results achieved by established wavelengths in state-of-the-art endodontics. |
| 45 | Ritter AL, Ritter AV, Murrah V, Sigurdsson A, Trope M. Pulp revascularization of replanted immature dog teeth after treatment with minocycline and doxycycline assessed by laser Doppler flowmetry, radiography, and histology. Dent Traumatol. 2004; 20: 75-84. | 91 | 4.55 (72) | Laser Doppler flowmetry (LDF) readings are beneficial in order to measure the vitality of teeth in regenerative treatments. |
| 46 | Rooney J, Midda M, Leeming J. A laboratory investigation of the bactericidal effect of a NdYAG laser. Br Dent J. 1994; 176: 61-4. | 91 | 3.03 (91) | At energy doses of 54 J and above, without black dye, a reduction of 10,000-fold or greater occurred. A similar reduction was achieved at energies above 25 J when black dye was added. These results indicate the energy levels which should be investigated to assess the potential role of the NdYAG laser in endodontics. |
| 48 | Chrepa V, Kotsakis GA, Pagonis TC, Hargreaves KM. The effect of photodynamic therapy in root canal disinfection: a systematic review. J Endod. 2014; 40: 891-8. | 90 | 9.00 (29) | Limited clinical information is currently available on the use of PDT in root canal disinfection. If supported by future clinical research, PDT may have efficacy for additional root canal disinfection, especially in the presence of multi-drug-resistant bacteria. |
| 49 | George R, Walsh LJ. Apical extrusion of root canal irrigants when using Er:YAG and Er,Cr:YSGG lasers with optical fibers: an in vitro dye study. J Endod. 2008; 34: 706-8. | 90 | 5.63 (56) | The volume of extruded fluid was similar to conventional 25-G needles, but fluid was distributed further from the apex. Because pulsed lasers create pressure waves in irrigant fluids within the root canal, the potential for extrusion of fluid from the apex should be considered when assessing intracanal laser treatments in endodontics. |
| 50 | Takeda FH, Harashima T, Kimura Y, Matsumoto K. Efficacy of Er:YAG laser irradiation in removing debris and smear layer on root canal walls. J Endod. 1998; 24: 548-51. | 90 | 3.46 (84) | These results show Er:YAG laser is effective in removing debris and smear layer from root canal walls. |
| 51 | Sgolastra F, Petrucci A, Gatto R, Monaco A. Effectiveness of laser in dentinal hypersensitivity treatment: a systematic review. J Endod. 2011; 37: 297-303. | 89 | 6.85 (44) | Laser therapy can reduce DH-related pain, but the evidence for its effectiveness is weak, and the possibility of a placebo effect must be considered. |
| 52 | Macedo RG, Wesselink PR, Zaccheo F, Fanali D, Van Der Sluis LW. Reaction rate of NaOCl in contact with bovine dentine: effect of activation, exposure time, concentration and pH. Int Endod J. 2010; 43: 1108-15. | 87 | 6.21 (52) | Activation is a strong modulator of the reaction rate of NaOCl. During the rest interval of 3min, the consumption of available chlorine increased significantly. This effect seems to be more pronounced after irrigant activation by laser. pH did not affect the reaction rate of 2% NaOCl. |
| 53 | Bergmans L, Moisiadis P, Teughels W, Van Meerbeek B, Quirynen M, Lambrechts P. Bactericidal effect of Nd:YAG laser irradiation on some endodontic pathogens ex vivo. Int Endod J. 2006; 39: 547-57. | 87 | 4.83 (67) | The Nd:YAG laser irradiation is not an alternative but a possible supplement to existing protocols for canal disinfection as the properties of laser light may allow a bactericidal effect beyond 1 mm of dentine. Endodontic pathogens that grow as biofilms, however, are difficult to eradicate even upon direct laser exposure. |
| 54 | Yang J, Yuan G, Chen Z. Pulp Regeneration: Current Approaches and Future Challenges. Front Physiol. 2016; 7: 58. | 86 | 10.75 (22) | This review focused on the requisite preconditions and cell homing strategies for pulp regeneration. In addition to the traditionally used mechanical preparation and irrigation, antibiotics, irrigation assisted with EndoVac apical negative-pressure system, and ultrasonic and laser irradiation are now being used in root canal disinfection. |
| 56 | Mehl A, Folwaczny M, Haffner C, Hickel R. Bactericidal effects of 2.94 microns Er:YAG-laser radiation in dental root canals. J Endod. 1999; 25: 490-3. | 85 | 3.40 (85) | As the results confirm, Er:YAG-laser radiation exerts very effective antimicrobial properties in dental root canals, depending on the time of radiation. |
| 55 | Wakabayashi H, Hamba M, Matsumoto K, Tachibana H. Effect of irradiation by semiconductor laser on responses evoked in trigeminal caudal neurons by tooth pulp stimulation. Lasers Surg Med. 1993; 13: 605-10. | 85 | 2.74 (92) | These results suggest that low power laser irradiation has a suppressive effect on injured tissue by blocking the depolarization of C-fiber afferents. |
| 60 | Alghaithy RA, Qualtrough AJ. Pulp sensibility and vitality tests for diagnosing pulpal health in permanent teeth: a critical review. Int Endod J. 2017; 50: 135-42. | 84 | 12.00 (15) | When accurately used and interpreted, pulp sensibility tests provide valuable diagnostic information, particularly when an electric pulp test is used in combination with either CO2 snow or Endo-Ice. |
| 57 | Ng R, Singh F, Papamanou DA, Song X, Patel C, Holewa C, Patel N, Klepac-Ceraj V, Fontana CR, Kent R, Pagonis TC, Stashenko PP, Soukos NS. Endodontic photodynamic therapy ex vivo. J Endod. 2011; 37: 217-22. | 84 | 6.46 (48) | Data indicate that PDT significantly reduces residual bacteria within the root canal system, and that PDT, if further enhanced by technical improvements, holds substantial promise as an adjunct to CMD. |
| 58 | Caviedes-Bucheli J, Ariza-García G, Restrepo-Méndez S, Ríos-Osorio N, Lombana N, Muñoz HR. The effect of tooth bleaching on substance P expression in human dental pulp. J Endod. 2008; 34: 1462-5. | 84 | 5.25 (59) | It was showed significant differences in the light (p<0.01) and laser (p<0.05) activated bleaching systems when compared with control values. It can be concluded that light- and laser-activated tooth-bleaching systems increase SP expression in human dental pulp significantly higher than normal values. |
| 59 | Liu HC, Lin CP, Lan WH. Sealing depth of Nd:YAG laser on human dentinal tubules. J Endod. 1997; 23: 691-3. | 84 | 3.11 (87) | Under SEM observation, nonlased specimens showed numerous exposed dentinal tubule orifices, and lased specimens showed melting of dentin and closure of exposed dentinal tubule orifices. The sealing depth of Nd:YAG laser on human dentinal tubules was approximately 4 microns. |
| 61 | Siddiqui SH, Awan KH, Javed F. Bactericidal efficacy of photodynamic therapy against Enterococcus faecalis in infected root canals: a systematic literature review. Photodiagnosis Photodyn Ther. 2013; 10: 632-43. | 82 | 7.45 (38) | Efficacy of PDT in eliminating E. faecalis from infected root canals remains questionable. |
| 62 | Wang QQ, Zhang CF, Yin XZ. Evaluation of the bactericidal effect of Er,Cr:YSGG, and Nd:YAG lasers in experimentally infected root canals. J Endod. 2007; 33: 830-2. | 82 | 4.82 (68) | Compared with the Er,Cr:YSGG laser, the Nd:YAG laser is more effective (p < 0.05). In conclusion, both lasers systems have a significant bactericidal effect in infected root canals, and the Nd:YAG laser is more effective than the Er,Cr:YSGG laser. |
| 63 | Ramsköld LO, Fong CD, Strömberg T. Thermal effects and antibacterial properties of energy levels required to sterilize stained root canals with an Nd:YAG laser. J Endod. 1997; 23: 96-100. | 82 | 3.04 (90) | Lasing cycles of 3 J-s for 15 s followed by a 15-s recovery interval can be continued for prolonged periods without risk of thermal damage to surrounding tissues. In vitro lasing of root canals inoculated with dark stained bacteria showed that two such lasing cycles sterilized only two out of eight canals, whereas when four cycles were used seven out of eight canals were sterilized. |
| 65 | Gordon W, Atabakhsh VA, Meza F, Doms A, Nissan R, Rizoiu I, Stevens RH. The antimicrobial efficacy of the erbium, chromium:yttrium-scandium-gallium-garnet laser with radial emitting tips on root canal dentin walls infected with Enterococcus faecalis. J Am Dent Assoc. 2007; 138: 992-1002. | 81 | 4.76 (69) | The results of this study suggest that the Er,Cr:YSGG laser with a radial emitting tip has a significant antimicrobial effect on dentinal tubules infected with E. faecalis. |
| 64 | Bonsor SJ, Nichol R, Reid TM, Pearson GJ. An alternative regimen for root canal disinfection. Br Dent J. 2006; 201: 101-5; discussion 98; quiz 20. | 81 | 4.50 (74) | Results indicate that the use of a chelating agent acting as a cleaner and disrupter of the biofilm and photo-activated disinfection to kill bacteria is an effective alternative to the use of hypochlorite as a root canal cleaning system. |
| 68 | Bergmans L, Moisiadis P, Huybrechts B, Van Meerbeek B, Quirynen M, Lambrechts P. Effect of photo-activated disinfection on endodontic pathogens ex vivo. Int Endod J. 2008; 41: 227-39. | 80 | 5.00 (61) | Photo-activated disinfection is not an alternative but a possible supplement to the existing protocols for root canal disinfection as the interaction between light (diode laser) and associated dye (TBO) provides a broad-spectrum effect. Some endodontic pathogens that grow as single-species biofilms, however, are difficult to eradicate. |
| 67 | Williams JA, Pearson GJ, Colles MJ. Antibacterial action of photoactivated disinfection {PAD} used on endodontic bacteria in planktonic suspension and in artificial and human root canals. J Dent. 2006; 34: 363-71. | 80 | 4.44 (76) | PAD killed endodontic bacteria at statistically significant levels compared to controls. Kills varied with bacterial species. |
| 66 | Moritz A, Schoop U, Goharkhay K, Jakolitsch S, Kluger W, Wernisch J, Sperr W. The bactericidal effect of Nd:YAG, Ho:YAG, and Er:YAG laser irradiation in the root canal: an in vitro comparison. J Clin Laser Med Surg. 1999; 17: 161-4. | 80 | 3.20 (86) | These experiments highlighted the potential of a carboxymethyl chitosan-based scaffold with growth factor releasing nanoparticles to promote migration and differentiation of SCAP. The results of this study may have direct application to improve current endodontic regenerative protocols. |
| 70 | Meire MA, Coenye T, Nelis HJ, De Moor RJ. Evaluation of Nd:YAG and Er:YAG irradiation, antibacterial photodynamic therapy and sodium hypochlorite treatment on Enterococcus faecalis biofilms. Int Endod J. 2012; 45: 482-91. | 78 | 6.50 (46) | Within the limitations of this particular laboratory set-up, NaOCl was the most effective in E. faecalis biofilm elimination, while Er:YAG laser treatment (100 mJ pulses) also resulted in high reductions in viable counts. The use of both commercial aPDT systems resulted in a weak reduction in the number of E. faecalis cells. Nd:YAG irradiation was the least effective. |
| 69 | Fonseca MB, Júnior PO, Pallota RC, Filho HF, Denardin OV, Rapoport A, Dedivitis RA, Veronezi JF, Genovese WJ, Ricardo AL. Photodynamic therapy for root canals infected with Enterococcus faecalis. Photomed Laser Surg. 2008; 26: 209-13. | 78 | 4.88 (65) | PDT was effective as a bactericidal agent in Enterococcus faecalis-contaminated root canals. |
| 71 | Smaïl-Faugeron V, Glenny AM, Courson F, Durieux P, Muller-Bolla M, Fron Chabouis H. Pulp treatment for extensive decay in primary teeth. Cochrane Database Syst Rev. 2018; 5: Cd003220. | 76 | 12.67 (12) | Lasers can be used in vital pulp treatments. |
| 72 | Xu Y, Young MJ, Battaglino RA, Morse LR, Fontana CR, Pagonis TC, Kent R, Soukos NS. Endodontic antimicrobial photodynamic therapy: safety assessment in mammalian cell cultures. J Endod. 2009; 35: 1567-72. | 75 | 5.00 (62) | The data suggest that there is a safe therapeutic window whereby PDT can inactivate endodontic pathogens without affecting host cell viability. |
| 73 | Afkhami F, Akbari S, Chiniforush N. Entrococcus faecalis Elimination in Root Canals Using Silver Nanoparticles, Photodynamic Therapy, Diode Laser, or Laser-activated Nanoparticles: An In Vitro Study. J Endod. 2017; 43: 279-82. | 74 | 10.57 (24) | PDT with ICG, an 810-nm diode laser, and AgNPs has the potential to be used as an adjunct for disinfection of the root canal system. |
| 74 | Bahcall J, Howard P, Miserendino L, Walia H. Preliminary investigation of the histological effects of laser endodontic treatment on the periradicular tissues in dogs. J Endod. 1992; 18: 47-51. | 73 | 2.28 (97) | At 30 days posttreatment, conventionally treated teeth began to return to normal morphology. The laser-treated teeth exhibited ankylosis, cemental lysis, and major bone remodeling. |
| 77 | Mainkar A, Kim SG. Diagnostic Accuracy of 5 Dental Pulp Tests: A Systematic Review and Meta-analysis. J Endod. 2018; 44: 694-702. | 72 | 12.00 (16) | LDF and PO were the most accurate diagnostic methods, and HPT was the least accurate diagnostic method. EPT showed high accuracy when testing vital teeth (specificity = 0.93) but low accuracy when assessing nonvital teeth (sensitivity = 0.72). CPT had moderate accuracy when evaluating vital (specificity = 0.84) and nonvital (sensitivity = 0.87) teeth. |
| 75 | Prażmo EJ, Kwaśny M, Łapiński M, Mielczarek A. Photodynamic Therapy As a Promising Method Used in the Treatment of Oral Diseases. Adv Clin Exp Med. 2016; 25: 799-807. | 72 | 9.00 (30) | Scientific data and published papers regarding the antibacterial properties of PDT will be subjected to analysis. Photodynamic therapy will be discussed as an alternative treatment protocol in oncology, endodontics, periodontology and other fields of dentistry. |
| 76 | Silva Garcez A, Núñez SC, Lage-Marques JL, Jorge AO, Ribeiro MS. Efficiency of NaOCl and laser-assisted photosensitization on the reduction of Enterococcus faecalis in vitro. Oral Surg Oral Med Oral Pathol Oral Radiol Endod. 2006; 102: e93-8. | 72 | 4.00 (81) | Laser photosensitization was effective for reducing E. faecalis in root canals and could be an adjunct to endodontic treatment. |
| 80 | Ordinola-Zapata R, Bramante CM, Aprecio RM, Handysides R, Jaramillo DE. Biofilm removal by 6% sodium hypochlorite activated by different irrigation techniques. Int Endod J. 2014; 47: 659-66. | 70 | 7.00 (42) | Laser activation of 6% sodium hypochlorite significantly improved the cleaning of biofilm-infected dentine followed by passive ultrasonic irrigation. |
| 78 | Garcez AS, Núñez SC, Azambuja N, Jr., Fregnani ER, Rodriguez HM, Hamblin MR, Suzuki H, Ribeiro MS. Effects of photodynamic therapy on Gram-positive and Gram-negative bacterial biofilms by bioluminescence imaging and scanning electron microscopic analysis. Photomed Laser Surg. 2013; 31: 519-25. | 70 | 6.36 (50) | The photodynamic effect seems to disrupt the biofilm by acting both on bacterial cells and on the extracellular matrix. |
| 79 | Zhang C, Wang X, Kinoshita J, Zhao B, Toko T, Kimura Y, Matsumoto K. Effects of KTP laser irradiation, diode laser, and LED on tooth bleaching: a comparative study. Photomed Laser Surg. 2007; 25: 91-5. | 70 | 4.12 (78) | The results of this study suggest that KTP laser is effective at providing brighter teeth. According to the conditions used in this study, the LED and KTP laser induced a safer pulpal temperature increase when assisted with Hi-Lite bleaching gel. |
| 81 | Moshonov J, Orstavik D, Yamauchi S, Pettiette M, Trope M. Nd:YAG laser irradiation in root canal disinfection. Endod Dent Traumatol. 1995; 11: 220-4. | 69 | 2.38 (94) | SEM analysis of teeth split longitudinally was used to illustrate the effect of treatment on the smear layer and on surface bacteria. Nd:YAG laser irradiation (group 3) significantly reduced the number of bacteria while NaOCl irrigation (group 6) effectively disinfected the canals. |
| 82 | Vohra F, Bukhari IA, Sheikh SA, Naseem M, Hussain M. Photodynamic activation of irrigation (using different laser prototypes) on push out bond strength of fiber posts. Photodiagnosis Photodyn Ther. 2020; 30: 101716. | 68 | 17.00 (6) | LAI with different laser prototypes improved push out bond values of PFRC post to root dentin as an adjunct to NaOCl and EDTA treatment. PDT improved push out strength compared to conventional canal cleaning regime. |
| 84 | Asnaashari M, Moeini M. Effectiveness of lasers in the treatment of dentin hypersensitivity. J Lasers Med Sci. 2013; 4: 1-7 | 68 | 6.18 (53) | Since a certain treatment has not yet introduced for dentin hypersensitivity, a combination of laser therapy and topical desensitizing factors ,can increase the success of the treatment compared with either treatments alone. |
| 83 | Meire MA, De Prijck K, Coenye T, Nelis HJ, De Moor RJ. Effectiveness of different laser systems to kill Enterococcus faecalis in aqueous suspension and in an infected tooth model. Int Endod J. 2009; 42: 351-9. | 68 | 4.53 (73) | The laser systems as well as PAD were less effective than NaOCl in reducing E. faecalis, both in aqueous suspension and in the infected tooth model. |
| 87 | Chiniforush N, Pourhajibagher M, Shahabi S, Bahador A. Clinical Approach of High Technology Techniques for Control and Elimination of Endodontic Microbiota. J Lasers Med Sci. 2015; 6: 139-50. | 65 | 7.22 (40) | Among different options, ICG could be the best choice due to its peak absorption at wavelength of 808 nm, which coincides with the commercial diode laser devices. Also, this wavelength has more penetration depth compared to other wavelengths used in aPDT. |
| 86 | Pedullà E, Genovese C, Campagna E, Tempera G, Rapisarda E. Decontamination efficacy of photon-initiated photoacoustic streaming (PIPS) of irrigants using low-energy laser settings: an ex vivo study. Int Endod J. 2012; 45: 865-70. | 65 | 5.42 (57) | Under the conditions of this ex vivo study, there were no significant differences in bacterial reduction between the laser and NaOCl or NaOCl alone groups. |
| 85 | Yilmaz HG, Kurtulmus-Yilmaz S, Cengiz E, Bayindir H, Aykac Y. Clinical evaluation of Er,Cr:YSGG and GaAlAs laser therapy for treating dentine hypersensitivity: A randomized controlled clinical trial. J Dent. 2011; 39: 249-54. | 65 | 5.00 (63) | Based on these findings, it may be concluded that both Er,Cr:YSGG and GaAlAs lasers were effective in the treatment of DH following a single application. |
| 88 | Heyeraas KJ, Kim S, Raab WH, Byers MR, Liu M. Effect of electrical tooth stimulation on blood flow, interstitial fluid pressure and substance P and CGRP-immunoreactive nerve fibers in the low compliant cat dental pulp. Microvasc Res. 1994; 47: 329-43. | 65 | 2.17 (99) | There was considerably less CGRP- and SP-immunoreactive fibers in the stimulated teeth than in the contralateral controls, suggesting that the vasodilation was caused by liberation of these sensory neuropeptides. |
| 89 | Silva LA, Novaes AB, Jr., de Oliveira RR, Nelson-Filho P, Santamaria M, Jr., Silva RA. Antimicrobial photodynamic therapy for the treatment of teeth with apical periodontitis: a histopathological evaluation. J Endod. 2012; 38: 360-6. | 64 | 5.33 (58) | Although apical closure by mineralized tissue deposition was not achieved, the absence of inflammatory cells, moderate neoangiogenesis, and fibrogenesis in the periapical region in the groups treated with aPDT indicate that this can be a promising adjunct therapy to cleaning and shaping procedures in teeth with apical periodontitis undergoing one-session endodontic treatment. |
| 90 | Miserendino LJ, Levy GC, Rizoiu IM. Effects of Nd:YAG laser on the permeability of root canal wall dentin. J Endod. 1995; 21: 83-7. | 64 | 2.21 (98) | The combined use of scanning electron microscopy and dye permeability measurements revealed a sealing of the dentinal wall by deposition of glass-like material and, in one specimen, the bridging of a lateral canal that partially occluded the canal. Based on statistical comparisons, permeability of laser-treated teeth was significantly less than untreated specimens |
| 91 | Peeters HH, Suardita K. Efficacy of smear layer removal at the root tip by using ethylenediaminetetraacetic acid and erbium, chromium: yttrium, scandium, gallium garnet laser. J Endod. 2011; 37: 1585-9. | 63 | 4.85 (66) | Use of a laser with a plain fiber tip, which produces cavitation in the irrigant, has potential as an improved alternative method for removing of the smear layer from the apical region of a straight root canal. |
| 92 | Moritz A, Jakolitsch S, Goharkhay K, Schoop U, Kluger W, Mallinger R, Sperr W, Georgopoulos A. Morphologic changes correlating to different sensitivities of Escherichia coli and enterococcus faecalis to Nd:YAG laser irradiation through dentin. Lasers Surg Med. 2000; 26: 250-61. | 62 | 4.13 (77) | Our study demonstrates the different morphologic impact of Nd:YAG laser irradiation through dentin on representatives of the two main groups of bacteria. It shows that the construction of the cell wall is crucial for their individual sensitivity to laser treatment. |
| 93 | Moritz A, Gutknecht N, Schoop U, Goharkhay K, Doertbudak O, Sperr W. Irradiation of infected root canals with a diode laser in vivo: results of microbiological examinations. Lasers Surg Med. 1997; 21: 221-6. | 62 | 2.58 (93) | Compared with the results achieved with the conventional bactericidal technique in the control group, the high power diode laser seems to be highly suitable for killing bacteria in infected root canals. |
| 95 | Abrar E, Naseem M, Baig QA, Vohra F, Maawadh AM, Almohareb T, et al. Antimicrobial efficacy of silver diamine fluoride in comparison to photodynamic therapy and chlorhexidine on canal disinfection and bond strength to radicular dentin. Photodiagnosis Photodyn Ther 2020; 32: 102066 | 62 | 15.25 (8) | Radicular dentin treated with 5.25 % NaOCl and 17 % EDTA displayed highest antimicrobial and bond strength scores. SDF and PDT can potentially be used in canal irrigation. |
| 96 | Schoop U, Goharkhay K, Klimscha J, Zagler M, Wernisch J, Georgopoulos A, et al. The use of the erbium, chromium:yttrium-scandium-gallium-garnet laser in endodontic treatment: the results of an in vitro study. J Am Dent Assoc 2007; 138: 949-55. | 61 | 3.59 (83) | The Er,Cr:YSGG laser can be used to eliminate bacteria in root canals. It also effectively removes smear layer and debris from the canal wall. |
| 97 | Takeda FH, Harashima T, Eto JN, Kimura Y, Matsumoto K. Effect of Er:YAG laser treatment on the root canal walls of human teeth: an SEM study. Endod Dent Traumatol 1998; 14: 270-3. | 61 | 2.35 (95) | The root canal walls irradiated by Er:YAG laser were free of debris, with an evaporated smear layer and open dentinal tubules. These results suggested that Er:YAG laser irradiation had an efficient cleaning effect on the prepared root canal walls. |
| 98 | Deleu E, Meire MA, De Moor RJ. Efficacy of laser-based irrigant activation methods in removing debris from simulated root canal irregularities. Lasers Med Sci 2015; 30: 831-5. | 61 | 6.56 (45) | Conventional irrigation removed significantly less debris than all other groups. The Er:YAG with plain fiber tip was more efficient than MDI, CI, diode, and Er:YAG laser with PIPS tip in removing debris from simulated root canal irregularities. |
| 99 | Wang X, Sun Y, Kimura Y, Kinoshita J, Ishizaki NT, Matsumoto K. Effects of diode laser irradiation on smear layer removal from root canal walls and apical leakage after obturation. Photomed Laser Surg 2005; 23: 575-81. | 59 | 3.11 (88) | These results indicate that the diode laser is useful for removing smear layer and debris from root canal walls, and reducing apical leakage after obturation in vitro, and suggest that it would be useful for root canal treatment in clinic. |
| 100 | Hardee MW, Miserendino LJ, Kos W, Walia H. Evaluation of the antibacterial effects of intracanal Nd:YAG laser irradiation. J Endod 1994; 20: 377-80. | 59 | 1.97 (100) | Analysis of the data indicated a 2-log reduction in colony-forming units among the four treatment groups as compared with the controls; however, no significant differences were observed among the treatment groups. In none of the treatment groups were the root canals sterilized. |
